# Closing in on a mechanism for activation

**DOI:** 10.7554/eLife.04909

**Published:** 2014-10-28

**Authors:** Stevan R Hubbard, W Todd Miller

**Affiliations:** 1 **Stevan R Hubbard** is in the Kimmel Center for Biology and Medicine at the Skirball Institute and the Department of Biochemistry and Molecular Pharmacology, New York University School of Medicine, New York, United Statesstevan.hubbard@med.nyu.edu; 2 **W Todd Miller** is in the Department of Physiology and Biophysics, Stony Brook University, Stony Brook, United Statestodd.miller@stonybrook.edu

**Keywords:** IGF1 receptor, mechanism, FRET, kinetics, human

## Abstract

When insulin-like growth factor-1 (IGF1) binds to its receptor, a physical constraint is released that allows the two transmembrane helices to come together to facilitate activation of the receptor.

**Related research article** Kavran JM, McCabe JM, Byrne PO, Connacher MK, Wang Z, Ramek A, Sarabipour S, Shan Y, Shaw DE, Hristova K, Cole PA, Leahy DJ. 2014. How IGF-1 activates its receptor. *eLife* **3**:e03772. doi: 10.7554/eLife.03772

**Image** Mechanism of receptor release upon ligand (insulin or IGF1) binding

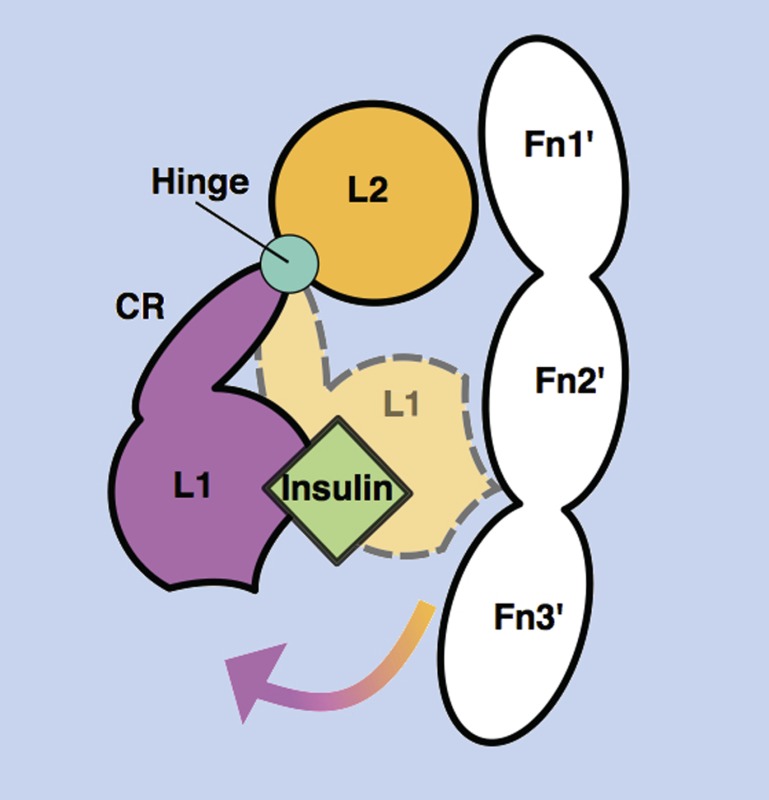

The hormone insulin and the related growth factor IGF1 (short for insulin-like growth factor-1) regulate the storage of fats and carbohydrates, growth and development, and many other important processes in human physiology (Nakae et al., 2001; De Meyts, 2004; Siddle, 2012). Nevertheless, precisely how these small proteins activate their receptors is still something of a mystery.

The receptors for insulin and IGF1 are members of the broad family of receptor tyrosine kinases. These proteins have an extracellular region that binds to the hormones, a segment that traverses the cell membrane (‘the transmembrane helix’), and an intracellular region that contains a tyrosine kinase domain (Figure 1). The kinase domain, when activated, adds phosphate groups to tyrosine residues on specific proteins. How a protein binding to the extracellular region activates the intracellular kinase domain has been the subject of over two decades of research (Lemmon and Schlessinger, 2010).

**Figure 1.**
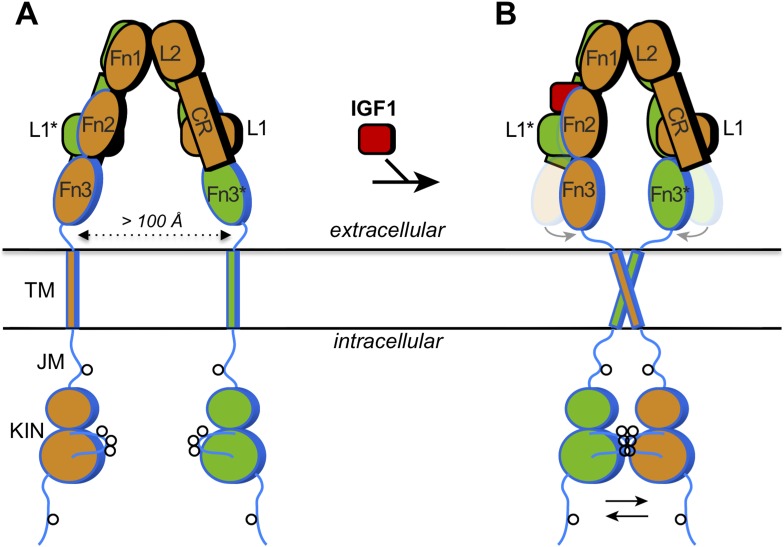
How IGF1 activates its receptor. Each IGF1 receptor is made of two half-receptors, which are linked by disulfide bonds (not shown). The six domains in the extracellular region of the first half-receptor (orange) are L1, CR, L2, Fn1, Fn2 and Fn3; the domains in the second half-receptor (green) are the same and labeled with an asterisk. The domains outlined in black are in the so-called α chain, and the domains outlined in blue are in the β chain of each half-receptor; the Fn2 domain consists of both chains. The intracellular region comprises the juxtamembrane region (JM) and the tyrosine kinase domain (KIN). Sites of *trans*-phosphorylation are shown as circles. (**A**) When IGF1 is not bound to the receptor, an interaction between L1* of the second half-receptor and Fn2 and Fn3 of the first half-receptor (and vice versa) is thought to maintain a large separation between the transmembrane (TM) helices (dotted arrow). (**B**) When IGF1 binds to L1* (or to L1), it disrupts the L1*-Fn2 (or L1-Fn2*) interaction. This allows Fn2 and Fn3 of each half-receptor to pivot (grey arrows) towards each other (the previous positions of Fn2 and Fn3 are shown semi-transparently). This in turn facilitates the dimerization of the TM helices in the membrane, which juxtaposes the kinase domains for efficient *trans*-phosphorylation (black arrows). Binding of a single IGF1 molecule (shown as binding to the left side) is sufficient to activate the receptor, but exactly how this asymmetry affects the conformational changes in the receptor is unclear.

For most proteins (or ligands) that are recognized by receptor tyrosine kinases, ligand binding to the receptor brings together two receptor proteins to work as a ‘dimer’ to send a signal into the cell. However, the insulin receptor and IGF1 receptor (together with a receptor known as ‘insulin receptor-related receptor’) are unique among receptor tyrosine kinases in that they are pre-formed into chemically (disulfide)-linked dimers. Therefore, their mode of activation must also be unique. Now in *eLife*, Daniel Leahy at the Johns Hopkins University School of Medicine and colleagues report new insights into how the insulin and IGF1 receptors are activated (Kavran et al., 2014).

Leahy and co-workers—who include Jennifer Kavran as the first author and others at Johns Hopkins and D E Shaw Research—have built on previous structural work on the insulin receptor. The crystal structure of the entire extracellular region of the insulin receptor was determined in the absence of insulin about a decade ago (McKern et al., 2006), while a structure of a fragment of the extracellular region that included bound insulin was published more recently (Menting et al., 2013).

The extracellular region of the insulin receptor forms an ‘inverted V’ shape, with a rather large distance (>100 Å) between the two transmembrane helices in the insulin-free state (Figure 1A). Kavran et al. demonstrated that the two kinase domains in the IGF1 receptor are activated by phosphorylating one another, extending the results of previous studies on the kinase domains alone (Favelyukis et al., 2001). Thus, these domains must be brought into close proximity when insulin or IGF1 binds. How does IGF1 stimulate this *trans*-phosphorylation? Is the IGF1 receptor constrained in some way that prevents the two kinase domains from associating with each other before IGF1 binds to the receptor? Or does binding of IGF1 stabilize the positioning of the two kinase domains side-by-side? Kavran et al. now provide evidence that IGF1 stimulates *trans*-phosphorylation by relieving a constraint within the extracellular region that holds the receptor in an inactive state (Kavran et al., 2014).

The two insulin structures mentioned above (McKern et al., 2006; Menting et al., 2013) do not fully reveal the structural changes that occur when insulin binds to the insulin receptor. However, these two structures suggested to Kavran et al. that a hinge movement between the so-called ‘cysteine-rich domain’ (CR in Figure 1) and the second ‘L domain’ (L2) might be involved. Based on this observation, Kavran et al. hypothesized that an interaction between the first L domain of one half-receptor and the second ‘fibronectin domain’ of the other half-receptor (L1* and Fn2 in Figure 1A) was needed to maintain the large separation between the transmembrane helices. They then proposed that IGF1 binding to L1* (its known primary binding site), disrupts the L1*-Fn2 interaction, which allows the transmembrane helices to approach one another (see Figure 1B).

Unfortunately, modified IGF1 receptors that contained amino acid substitutions designed to disrupt the interaction between L1* and Fn2 were poorly expressed in cells, meaning that Kavran et al. could not test this interaction's importance in this manner. However, deleting the entire L1 domain did the trick, and revealed that this truncated IGF1 receptor was always switched on—even when IGF1 was absent. This supports the idea that the L1*-Fn2 interaction inhibits the receptor's activity. Also consistent with this hypothesis, when Kavran et al. included flexible linkers between the Fn3 domain and the transmembrane helix, there was greater phosphorylation of the intracellular kinase domains in the absence of IGF1.

Kavran et al. then replaced the intracellular portion of the IGF1 receptor with fluorescent probes, and used a technique called Förster resonance energy transfer (FRET) to measure the distance between the transmembrane helices, both with and without IGF1. These data supported the notion that the transmembrane helixes get closer when IGF1 binds. But how close together do they get? Further biochemical studies and computer simulations showed that the transmembrane helices probably dimerize, or physically interact, which would help position the kinase domains next to one another for *trans*-phosphorylation.

So, what more is there to learn about the activation of the insulin and IGF1 receptors? Another recent study has proposed that, when the hormone binds, the insulin receptor is activated by the transmembrane helices moving away from each other (Lee et al., 2014). This is essentially the opposite mechanism to that proposed by Kavran et al. Further work is needed to resolve these conflicting models of activation.

Other key questions remain unanswered. In particular, how do IGF1 and insulin bind to their secondary, ‘crosslinking’ sites on the receptors (Fn1 and Fn2 in Figure 1B), and how are the kinase domains arranged in the inactivated and activated states? Though the transmembrane helices may be far apart in the inactive insulin receptor (or IGF1 receptor), the juxtamembrane region (JM in Figure 1A) is rather long. Therefore, if this region were not constrained, the two kinase domains should be capable of reaching one another (for *trans*-phosphorylation). The nature of this steric constraint is unknown. Finally, Kavran et al. observed that the presence of the juxtamembrane region increased kinase activity fourfold relative to the kinase domains alone. Thus, the juxtamembrane regions of the IGF1 receptor and insulin receptor hold mechanistic secrets yet to be revealed.
